# Effectiveness of negative pressure wound therapy with instillation and dwelling after stoma closure: a retrospective and propensity score matching analysis

**DOI:** 10.1038/s41598-022-05016-1

**Published:** 2022-01-18

**Authors:** Yoshinori Yane, Jin-ichi Hida, Yasutaka Chiba, Yusuke Makutani, Hokuto Ushijima, Yasumasa Yoshioka, Masayoshi Iwamoto, Toshiaki Wada, Koji Daito, Tadao Tokoro, Kazuki Ueda, Junichiro Kawamura

**Affiliations:** 1 Department of Surgery, Faculty of Medicine, Kindai University, 377-2 Ohno-Higashi, Osaka-sayama, Osaka 589-8511 Japan; 2 Department of Gastroenterological Surgery, Kindai University Nara Hospital, Ikoma, Nara Japan; 3 Division of Biostatistics, Clinical Research Center, Faculty of Medicine, Kindai University, Osaka-sayama, Osaka Japan

**Keywords:** Gastrointestinal system, Anal cancer, Colorectal cancer, Gastroenterology, Gastrointestinal diseases, Inflammatory bowel disease

## Abstract

The use of temporary diverting stoma has become more common in low colorectal anastomosis to reduce anastomotic complications. Surgical site infection (SSI) at the stoma closure site has been one of the most frequent postoperative complications. The aim of this study was to compare the short-term outcomes between conventional primary suture closure and negative pressure wound therapy with instillation and dwelling (NPWTi-d) therapy following purse-string suturing, using propensity score matching analysis. We retrospectively evaluated the medical records of 107 patients who underwent stoma closure between January 2016 and October 2020. The primary outcome was the proportion of SSI. The secondary outcome was the day of postoperative length of stay. Propensity score matching with one-to-one match was performed for reducing treatment selection bias. Of a total of 107 patients, 67 patients had been treated with conventional primary closure and 40 with NPWTi-d therapy. The propensity score matching derived 37 pairs. The respective SSI proportions were 0% and 16.2% in the groups with NPWTi-d and primary closure (*P* = 0.025). The respective median days of postoperative hospital stay were 9.0 and 10.0 in the groups with NPWTi-d and primary closure (*P* = 0.453). NPWTi-d therapy with purse-string suturing was effective in reducing SSI after stoma closure.

## Introduction

The use of temporary diverting stoma has become more common in low colorectal anastomosis to reduce anastomotic complications^1^. Stoma closure is a relatively easy operation; however, one of the most frequent postoperative complications is surgical site infection (SSI) after stoma closure. SSI following stoma closure increases the patient efforts and psychological stress, as well as resulting in a longer hospital stay, more frequent outpatient follow-ups, and greater medical costs^2^. In addition, an abdominal incisional hernia may develop as a late complication of SSI after stoma closure^3^.

Primary suturing has often been used to close the skin at stoma closure site; however, the incidence of SSI is remarkably high^4^. Since Banarjee reported a wound closure method with purse-string suture, the method has been generally used as a preventive measure^5,6^. Alternatively, negative pressure wound therapy (NPWT) may be used in recent years^7,8^. NPWT is a wound dressing system that continuously apply subatmospheric pressure to the system, which draws out fluid and promotes angiogenesis, reduces oedema, increases tensile strength, and reduces SSI. However, the effectiveness of using NPWT for stoma closure wounds has not yet been demonstrated. Purse-string skin closure is very effective in preventing SSI^6^; however, epithelialisation of the wound takes a long time^9^. NPWT is very useful for granulation. However, local infections may occur because NPWT itself creates a closed space^7^.

The use of NPWT with instillation and dwelling (NPWTi-d) has been of current interest. NPWTi-d has the benefits of automatic cleaning of the wound surface and dissolving of devitalised tissue. It can aggressively remove exudate and can decrease the bacterial load. NPWTi-d has been shown to be effective for contaminated wounds, however, its use and effectiveness for stoma closures has not been proven.

The aim of this study was to compare the short-term outcomes between conventional primary suture closure and NPWTi-d therapy with purse-string suturing using propensity score matching analysis.

## Methods

### Patients and enrolment criteria

From January 2016 to October 2020, a total of 107 patients underwent surgery for stoma closure in our hospital. Patients were divided into two groups according to operative management. From January 2016 to November 2018, all skin wounds were closed by conventional primary closure. From December 2018 to October 2020, all skin wounds were closed by purse-string closure and managed using NPWTi-d.

The inclusion criteria were as follows: patients who underwent stoma closure using conventional primary closure or NPWTi-d therapy. Written informed consent was obtained from all patients before study enrolment. The exclusion criteria were as follows: patients aged less 15 years old; patients who could not be tracked in medical records. Written informed consent was obtained from all individual participants included in the study. In the case of participants who were minors, informed consent was obtained from a parent or legal guardian. The study was conducted in accordance with the Declaration of Helsinki on human subjects. Further, this study was approved by the Institutional Ethical Review Board of Kindai University (approval no. R02-181) and registered at the UMIN Clinical Trials Registry as UMIN000044424, 04/06/2021 (http://www.umin.ac.jp/ctr/index-j.htm).

### Surgical technique

**Figure 1:**
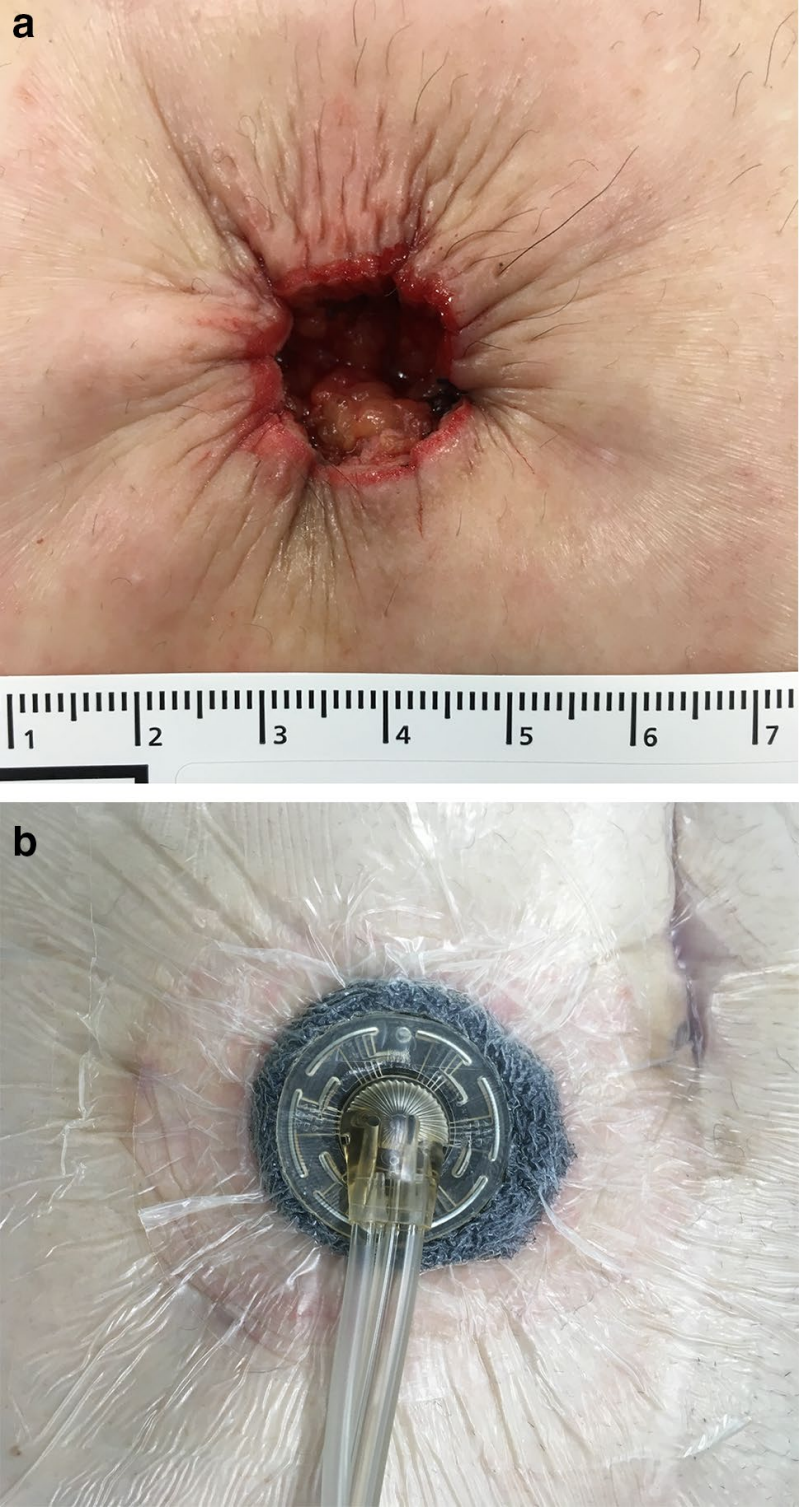
(**a**) Fascial closure was performed to an interrupted manner with an 0 polydioxanone suture (PDS). The skin was closed by using a purse-string subcuticular continuous suture with 4–0 PDS, leaving an open 10–20 mm circular gap. (**b**) V.A.C. VERAFLO was attached to the wound.

All patients were administered general anaesthesia. For the patients who underwent stoma closure by primary closure, a spindle-shaped skin incision was made in the craniocaudal direction. For the patients who were treated with NPWTi-d, a round skin incision was made. In both groups, stoma reversal was performed using the same procedure. The subcutaneous tissue around the stoma was incised, and adhesions around the intestine were detached from the abdominal wall. After the intestine had been mobilised, the segment that remained outside the abdominal wall was excised. A functional end-to-end anastomosis or Albert-Lembert anastomosis was then created. Glove change was performed, and a new set of instruments was used after anastomosis. Interrupted sutures (0 polydioxanone, PDS, Ethicon, Cincinnati, Ohio, USA) were used for fascial closure. After confirming haemostasis, the subcutaneous tissue was washed with 1500 ml of physiological saline. In the primary closure group, the buried suture was performed with 4-0 PDS (polydioxanone, PDS, Ethicon, Cincinnati, Ohio, USA). In the NPWTi-d group, the skin was closed using a purse-string subcuticular continuous suture with 4-0 PDS, leaving a 10–20 mm open circular gap (Fig. 1a). In addition, NPWTi-d (V.A.C. VERAFLO Therapy, KCI, an Acelity Company, San Antonio, Texas) was attached to the wound (Fig. 1b).

### Preoperative and postoperative care

According to our hospital protocol, all patients who underwent stoma closure received mechanical preparation with oral laxatives. Antibiotic prophylaxis, 1.0 g flomoxef sodium (FLUMARIN, Shionogi & Co., LTD, Osaka, Japan) was administered 30 min before the incision was made. The first drink was administered on postoperative day 1 (POD1), and the first solid oral intake was on POD3.

### NPWTi-d therapy

**Figure 2:**
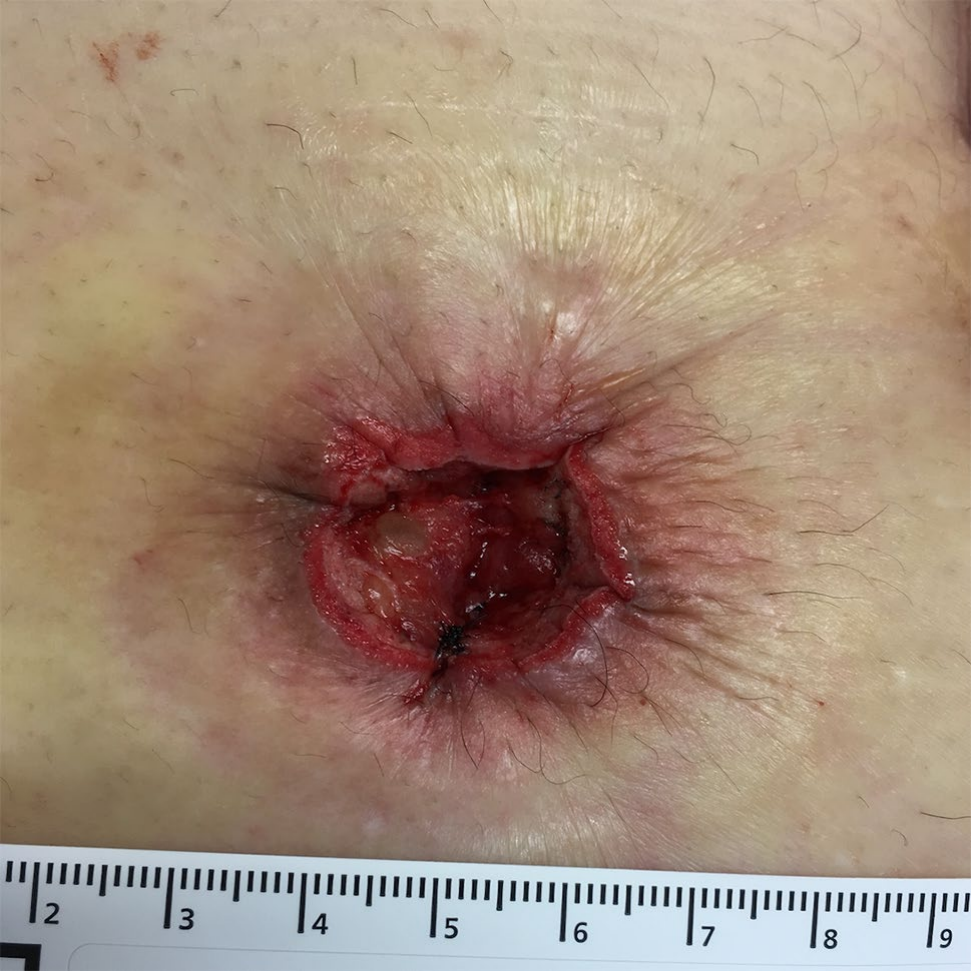
The granulation had grown to the extent that the abdominal rectus muscle fascia and the suture was covered. There was no obstruction of blood flow in the granulation. NPWTi-d therapy was finished.

**Figure 3:**
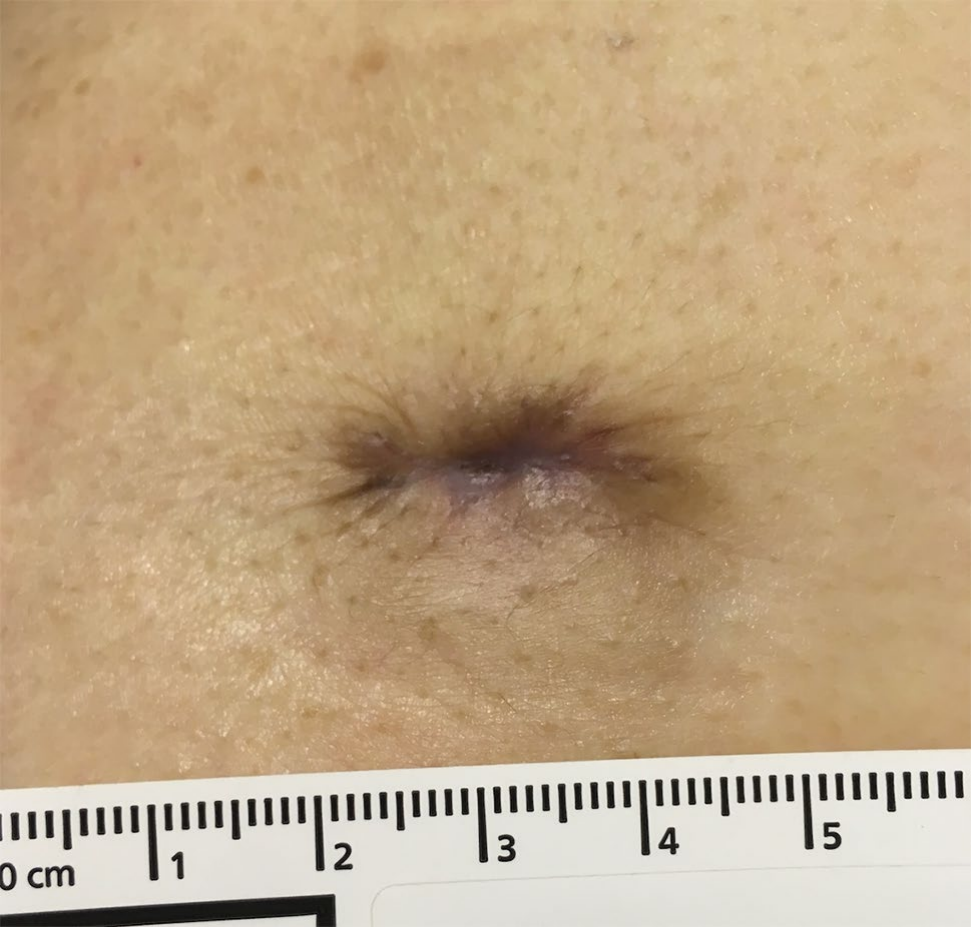
The epithelialisation was confirmed.

Immediately after surgery, NPWTi-d was attached to the wound and treatment was started. The V.A.C. VERAFLO settings were set to an instill volume of 2 ml, soak time of 2 min, and a V.A.C. therapy time of 2 h. The target pressure was − 75 mmHg, and the intensity was low. The foam was removed on POD 3 or 4. If it was confirmed that the granulation had covered the abdominal rectus muscle fascia and the sutures, NPWTi-d therapy was completed (Fig. 2). If the granulation did not cover the abdominal rectus muscle fascia, NPWTi-d therapy was continued for another 3 days or so. After that, the wound was washed with physiological saline once a day and treated with gentamicin ointment. The patients were discharged if they were able to eat. After discharge, patients performed the procedure themselves by cleansing the wound in the shower followed by treatment with gentamicin ointment. Once epithelialisation was confirmed, the outpatient visits were terminated (Fig. 3).

### Outcome measures

The primary endpoint was the rate of postoperative SSI. SSI was defined according to the Center of Disease Control and Prevention (CDC) criteria and includes both superficial or deep incisional SSI^10^. In summary, superficial incisional SSIs involve only skin and subcutaneous tissue within 30 days of the operation. Deep incisional SSIs involve the muscle and fascial layers but not the organ space. Diagnosis of SSIs was performed by the surgeon or attending physicians. The secondary endpoint was the length of postoperative hospital stay after surgery. Adverse events were evaluated using the Common Terminology Criteria for Adverse Events (CTCAE) v.0.4.0. Postoperative complications were evaluated based on the classification of Clavian-Dindo.

### Statistical analysis

Propensity score matching was performed for reducing treatment selection bias and increasing comparability between the primary closure and NWPTi-d therapy groups. For the matching, four factors of body mass index (BMI), chemotherapy, diabetes mellitus, and stoma location were used as covariates. The matching ratio was set to one-to-one, where a caliper width of 0.2 was used without replacement. Analyses were performed using unpaired methods rather than paired methods.

The proportion of postoperative SSI, which was the primary endpoint, was yielded, and the comparison between the two treatment groups was made using Fisher’s exact test. For the days of postoperative hospital stay after surgery, which was the secondary endpoint, medians and ranges were yielded and the comparison between the two treatment groups was made using Mann–Whitney *U* test.

For the other outcomes and baseline characteristics, frequencies and percentages were applied for categorical variables with Fisher’s exact test for the comparison between the two treatment groups, while the medians and ranges were applied for continuous variables with Mann–Whitney *U* for the comparison between the two treatment groups.

Statistical analyses were performed using EZR software (Saitama Medical Center, Jichi Medical University, Saitama, Japan), which is a graphical user interface for R (The R Foundation for Statistical Computing, Vienna, Austria)^11^.

## Results

### Demographics and clinical characteristics

**Figure 4:**
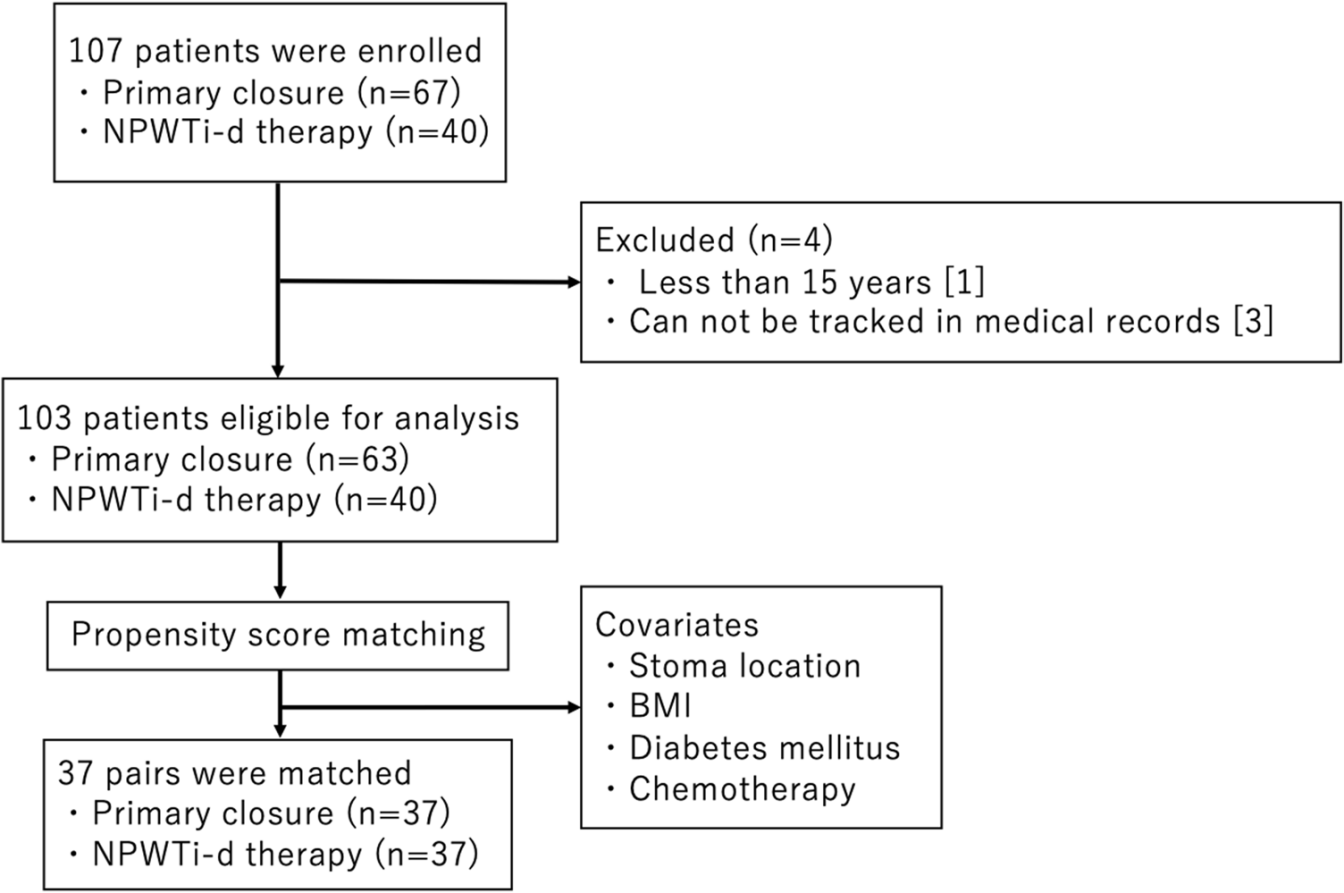
Flowchart of the patients selection.

Between January 2016 and October 2020, we performed 107 stoma closures in our hospital. Among them, 67 patients underwent conventional primary closure and 63 patients were eligible for inclusion in the study. Forty patients underwent NPWTi-d therapy and were eligible. Under the propensity score matching, 37 pairs were matched and analysed (Fig. 4).

Table 1 presents the clinical characteristics between the two groups before and after propensity score matching. Before propensity score matching, there were no significant differences in age, sex, BMI, American Society of Anesthesiologists (ASA) score, albumin level, smoking history, primary disease, stoma location, chemotherapy, use of steroids, and diabetes mellitus between the two groups. Even under propensity score matching, there was no significant difference between the two groups.

**Table 1:** Characteristics of the patients before and after propensity score matching.

	Before propensity score matching			After propensity score matching		
	Primary closure	NPWTi-d therapy	*P* value	Primary closure	NPWTi-d therapy	*P* value
	(n = 63)	(n = 40)		(n = 37)	(n = 37)	
Sex (male/female)	45/18	29/11	1.000	28/9	26/11	0.794
Age, years	66 (19–87)	66 (19–83)	0.689	69 (20–87)	66 (19–83)	0.153
BMI	21.8 (15.0–30.9)	21.5 (15.6–36.9)	0.768	21.8 (15.0–29.3)	21.6 (15.6–36.9)	0.863
Alb	4.1 (2.9–5.0)	4.1 (2.6–4.6)	0.582	4.1 (2.9–5.0)	4.0 (2.6–4.6)	0.497
**ASA**			0.689			0.829
I	6 (9.5%)	2 (5.0%)		4 (10.8%)	2 (5.4%)	
II	51 (81.0%)	33 (82.5%)		29 (78.4%)	30 (81.1%)	
III	6 (9.5%)	5 (12.5%)		4 (10.8%)	5 (13.5%)	
**Primary disease**			0.288			0.122
Cancer	45 (71.4%)	30 (75.0%)		24 (64.9%)	28 (75.7%)	
Ulcerative colitis	7 (11.1%)	5 (12.5%)		3 (8.1%)	5 (13.5%)	
Other	11 (17.5%)	5 (12.5%)		10 (27.0%)	4 (10.8%)	
**Stoma location (%)**			0.791			1.000
Ileum	51 (81.0%)	34 (85.0%)		32 (86.5%)	32 (86.5%)	
Colon	12 (19.0%)	6 (15.0%)		5 (13.5%)	5 (13.5%)	
Steroid use (%)	7 (11.1%)	5 (12.5%)	1.000	4 (10.8%)	5 (13.5%)	1.000
Smoking (%)	20 (31.7%)	15 (37.5%)	0.670	13 (35.1%)	14 (37.8%)	1.000
DM (%)	7 (11.1%)	7 (17.5%)	0.388	4 (10.8%)	4 (10.8%)	1.000
Chemotherapy (%)	30 (47.6%)	17 (42.5%)	0.687	17 (45.9%)	17 (45.9%)	1.000

*BMI*, Body mass index; *Alb*, Albumin; *ASA*, American Society of Anesthesiologists; *DM*, diabetes mellitus, *NPWTi-d*, negative pressure wound therapy with instillation and dwelling.

### Postoperative outcomes

Table 2 presents the data on postoperative outcomes between the two groups. There were no significant differences after propensity score matching in the median operative time (89.0 vs 88.0 min, *P* = 0.721), blood loss (10.0 vs 10.0 ml, *P* = 0.656). The median postoperative hospital stay was not significantly different between the two groups (10 vs 9 days, *P* = 0.453).

**Table 2:** Postoperative outcomes.

	Before propensity score matching			After propensity score matching		
	primary closure	NPWTi-d therapy	P value	primary closure	NPWTi-d therapy	P value
	(n = 63)	(n = 40)		(n = 37)	(n = 37)	
Operative time (min)	91.0 (47.0–173.0)	90.0 (56.0–152.0)	0.986	89.0 (60.0–154.0)	88.0 (56.0–152.0)	0.721
Blood loss (ml)	10.0 (5.0–275.0)	10.0 (5.0–110.0)	0.429	10.0 (5.0–275.0)	10.0 (5.0–110.0)	0.656
**Complications (%)**						
SSI	7 (11.1%)	0 (0.0%)	0.041	6 (16.2%)	0 (0.0%)	0.025
Ileus	1 (1.6%)	2 (5.0%)	0.558	1 (2.7%)	1 (2.7%)	1.000
Diarrhea	1 (1.6%)	1 (2.5%)	1.000	1 (2.7%)	1 (2.7%)	1.000
Cerebral infarction	1 (1.6%)	0 (0.0%)	1.000	1 (2.7%)	0 (0.0%)	1.000
Infection	0 (0.0%)	1 (2.5%)	0.388	0 (0%)	1 (2.7%)	1.000
Postoperative hospital stay (days)	9.0 (5.0–43.0)	9.0 (6.0–18.0)	0.703	10.0 (5.0–43.0)	9.0 (6.0–18.0)	0.453

*SSI*, Surgical site infection.

### Postoperative surgical site infection

Before propensity score matching, 7 patients (11.1%) developed SSI in the primary closure group and no patient developed SSI (0%) in the NPWTi-d therapy group (*P* = 0.041). After propensity score matching, 6 patients (16.2%) developed SSI in the primary closure group and no patient (0%) developed SSI in the NPWTi-d therapy group. The NPWTi-d therapy group had a significantly lower SSI rate than the primary closure group (*P* = 0.025).

### Short-term complications

Data regarding postoperative complications are shown in Table 2. Before propensity score matching, one patient (1.6%) presented with ileus, one patient (1.6%) developed diarrhoea, and one patient (1.6%) had cerebral infarction (Clavien-Dindo classification ≥ Grade 2) in the primary closure group. Two patients (5%) presented with ileus, one patient (2.5%) developed diarrhoea, and one patient (2.5%) developed infection (Clavien-Dindo classification ≥ Grade 2) in the NPWTi-d therapy group.

After propensity score matching, there was no significant difference between the primary closure group and NPWTi-d therapy group in the rates of ileus (2.7% vs. 2.7%, *P* = 1.000), cerebral infarction (2.7% vs 0%, *P* = 1.000), infection (0% vs. 2.7%, *P* = 1.000), and diarrhoea (2.7% vs. 2.7%, *P* = 1.000).

## Discussion

From this study, we found that stoma closure using NPWTi-d was an effective treatment for stoma wound closure. No patients developed SSI when using NPWTi-d, and the management was significantly better than conventional primary suture closure.

A temporary stoma is usually used for the patients undergoing a low pelvic anastomosis with rectal cancer and benign diseases, such as ulcerative colitis, Crohn’s disease, and familial adenomatous polyposis. The most unfavourable complication of a low pelvic anastomosis is anastomotic leak^12–14^. The temporary diverting stoma may be able to avoid an anastomotic leak.

Complications after stoma closure include SSI, intestinal obstruction, incisional hernia, and anastomotic leakage. SSI is the most common surgical complication after stoma closure. Since the procedure requires enteric anastomosis, stoma closure is a clean-contaminated procedure. Risk factors for SSI include chemotherapy, obesity, diabetes mellitus, history of smoking, long-term steroid administration, and immunosuppressant administration^15,16^. In addition, the type of stoma created affects the incidence of SSIs. Colostomy has a higher incidence of SSI than ileostomy. A previous study reported that colostomy reversal was associated with a fivefold increase in SSI compared with ileostomy reversal. It is thought that this may be because the colon tends to harbour a higher bacterial count and be associated with an increased risk of SSI^17^. SSI leads to an increase in the treatment burden on medical staff and patients. It causes longer postoperative hospital stays, more outpatient visits, additional home health care utilization, and higher health care costs. In addition, an abdominal incisional hernia may develop as a late complication of SSI after stoma closure. An abdominal incisional hernia can significantly reduce the patient's quality of life and, in some cases, may require surgical procedures. Thus, it imposes an increased burden on the patient and increases medical costs^15^.

Several treatments have been attempted to reduce SSI after stoma closure, and several studies have reported that primary closure is linked with high rates of SSI, with reported rates vary between 2 and 40%^4,18–20^. In primary suture closure, the wound closes immediately and dead space may form, in which subcutaneous fluid accumulates and an abscess may form^21^. A few studies reported primary closure with drainage tubes being placed in the subcutaneous layer below the wound. However, this technique still has a high infection rate of approximately 20%^22^.

Purse-string sutures have one of the lowest infection rates, and have proven to be a useful technique^2,5,22^. However, this method often takes a long time to complete granulation and epithelialization^23^. Patients require continuous wound care and outpatient visits. Elderly patients may have difficulty performing self-care treatment.

NPWT therapy can compensate for these problems. Currently, although NPWT is used in a variety of wounds, its prophylactic use is not yet considered essential after stoma closure. A study on the use of NPWT in preventing SSIs and improving wound healing time after stoma closure has failed to demonstrate the efficacy of NPWT in comparison to purse-string sutures^7^. Local infection still occurs with NPWT^7,24^.

NPWTi-d therapy may be able to further reduce infection rates. NPWTi-d can prevent bacterial growth by automatic cleansing of the wound surface as well as early and thorough removal of dissolving devitalised tissue and exudate. By using NPWTi-d on the stoma closure wound, the promotion of granulation may reduce the dead space and the risk of SSI^25–31^. It may also shorten the wound healing period. This will reduce the burden on medical staff and patients and lead to a reduction in inpatient duration and outpatient visits. However, it has not been clarified whether NPWTi-d is useful for prophylactic use of SSI after stoma closure. In this study, NPWTi-d therapy was remarkably useful for SSI after stoma closure. On the other hand, we could not prove the usefulness of shortening the length of hospital stay and shortening the healing period. The combined use of NPWTi-d therapy and delayed primary closure may be useful as a solution to these problems^32^.

NPWT therapy is often associated with the higher-cost advanced would care therapies. Some studies on NPWT therapy for the patients with a wide range of surgeries have not proven to be cost-effective compared to conventional simple closure and purse-string wound closure^33^. Although NPWTi-d therapy might be reported potentially cost-effective than NPWT therapy in a hypothetical economic model, the question remains whether NPWT therapy could achieve cost saving for patients after stoma closure^34^. Therefore, further study is warranted to assess the comparative cost-effectiveness of each treatment.

This study has several limitations. First, this was a single-centre retrospective study; thus, the number of patients was limited. Second, it was a comparison between NPWTi-d therapy and conventional primary closure, not a purse-string wound closure or NPWT therapy. Third, this study did not include the patients who underwent stoma closure after Hartmann procedure since there were no patients during the study observation period. NPWTi-d therapy might be much effective for the patients who underwent stoma closure after Hartmann procedure^17^.

In conclusion, NPWTi-d therapy at the stoma closure site was a highly effective treatment for reducing SSI. We believe that NPWTi-d therapy should always be considered at stoma closure site. Further ingenuity will be needed to shorten the length of hospital stay and the healing period of wounds.
